# Supplementary material for: First Report of Complete Mitochondrial Genome in the Tribes Coomaniellini and Dicercini (Coleoptera: Buprestidae) and Phylogenetic Implications
Source: Genes (Basel). 2022 Jun 16;13(6):1074. doi: 10.3390/genes13061074 (PMC9222259; doi:10.3390/genes13061074)
Supplement: Supplementary file 1 [file genes-13-01074-s001.zip › genes-1739171-supplementary.pdf]

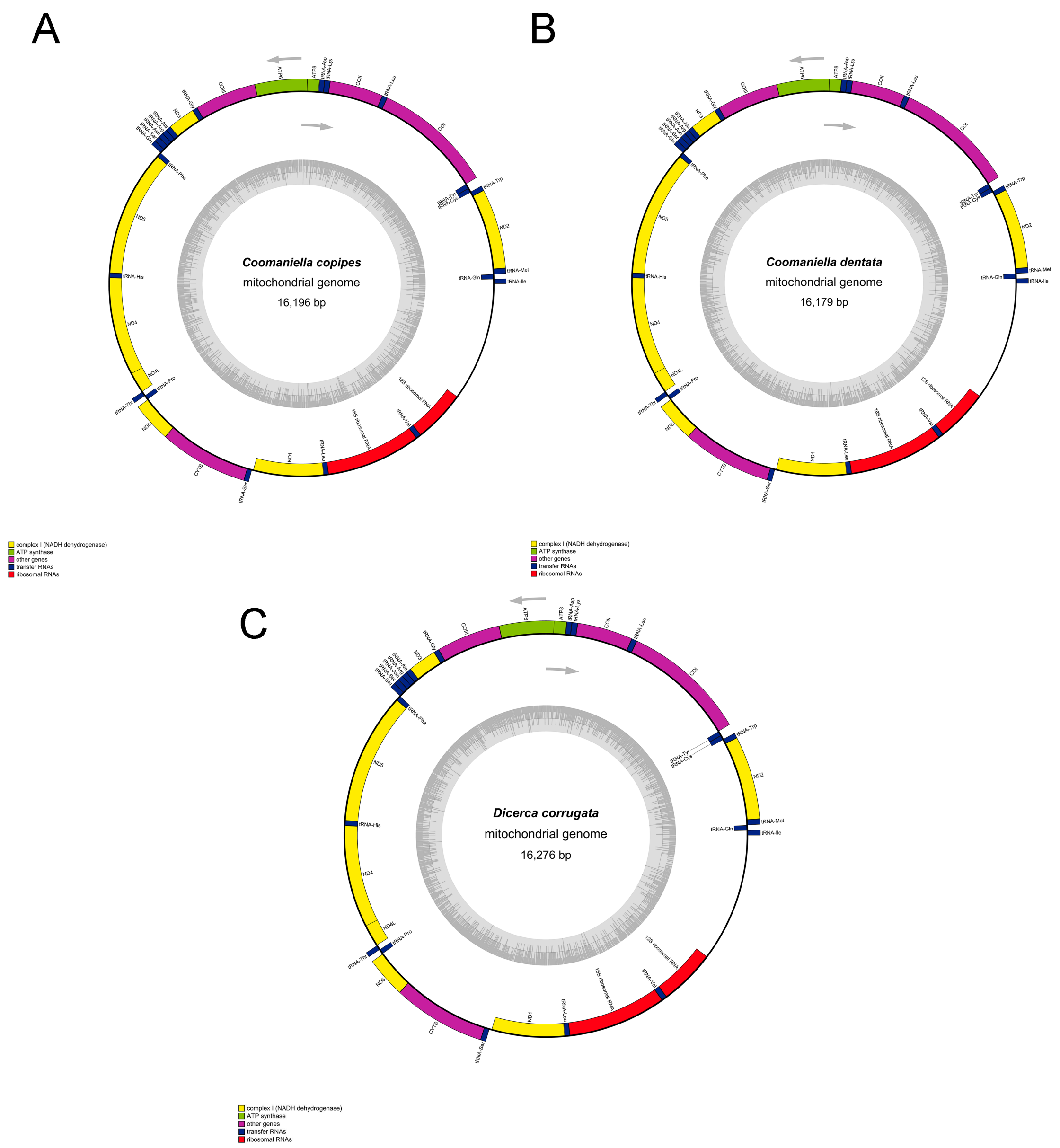

Figure S1. The mitogenome maps of *Coomaniella copipes* (**A**), *Coomaniella dentata* (**B**) and *Dicerca corrugata* (**C**).

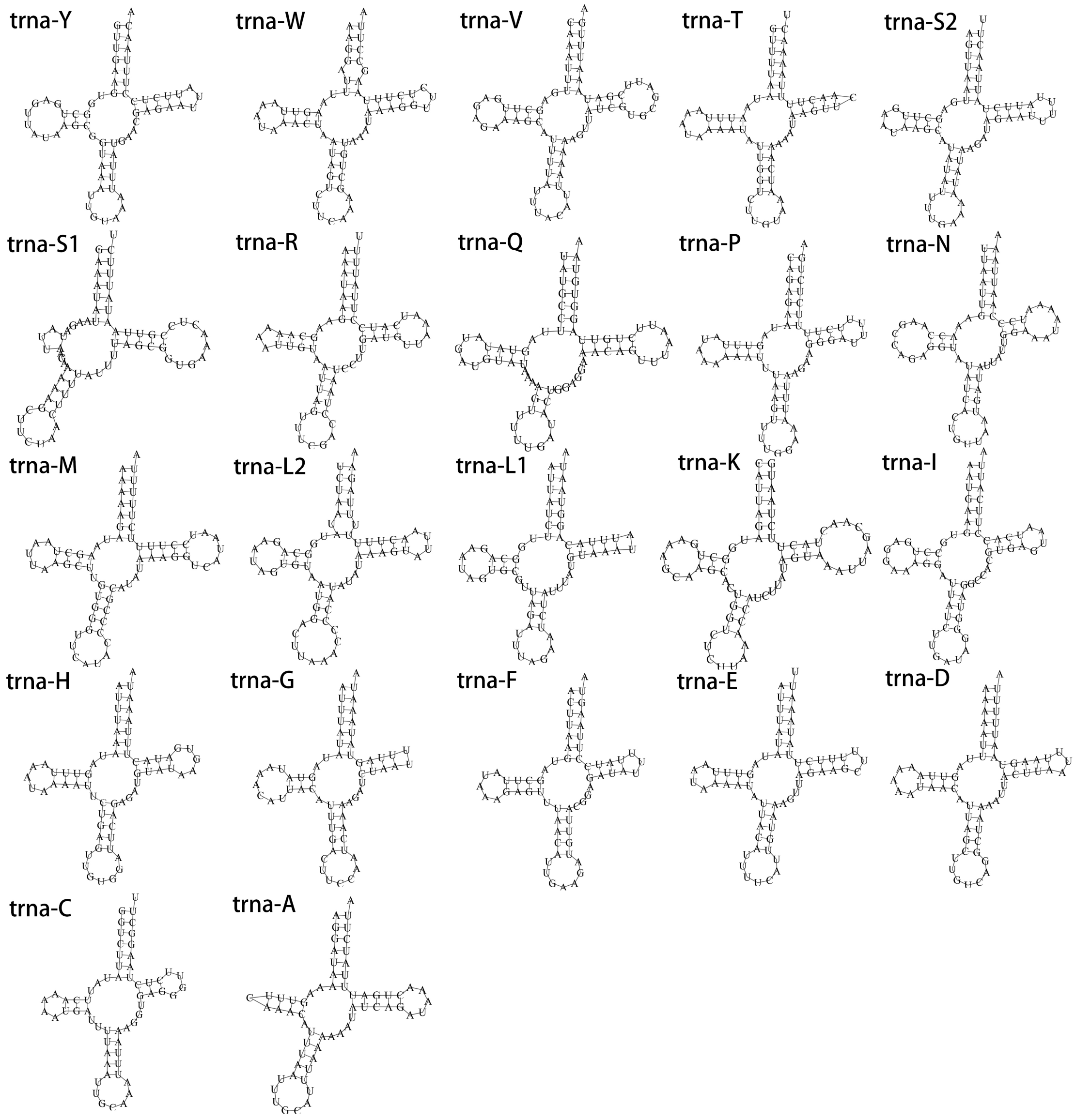

Figure S2. The secondary cloverleaf structure for the tRNAs of *Coomaniella copipes*.

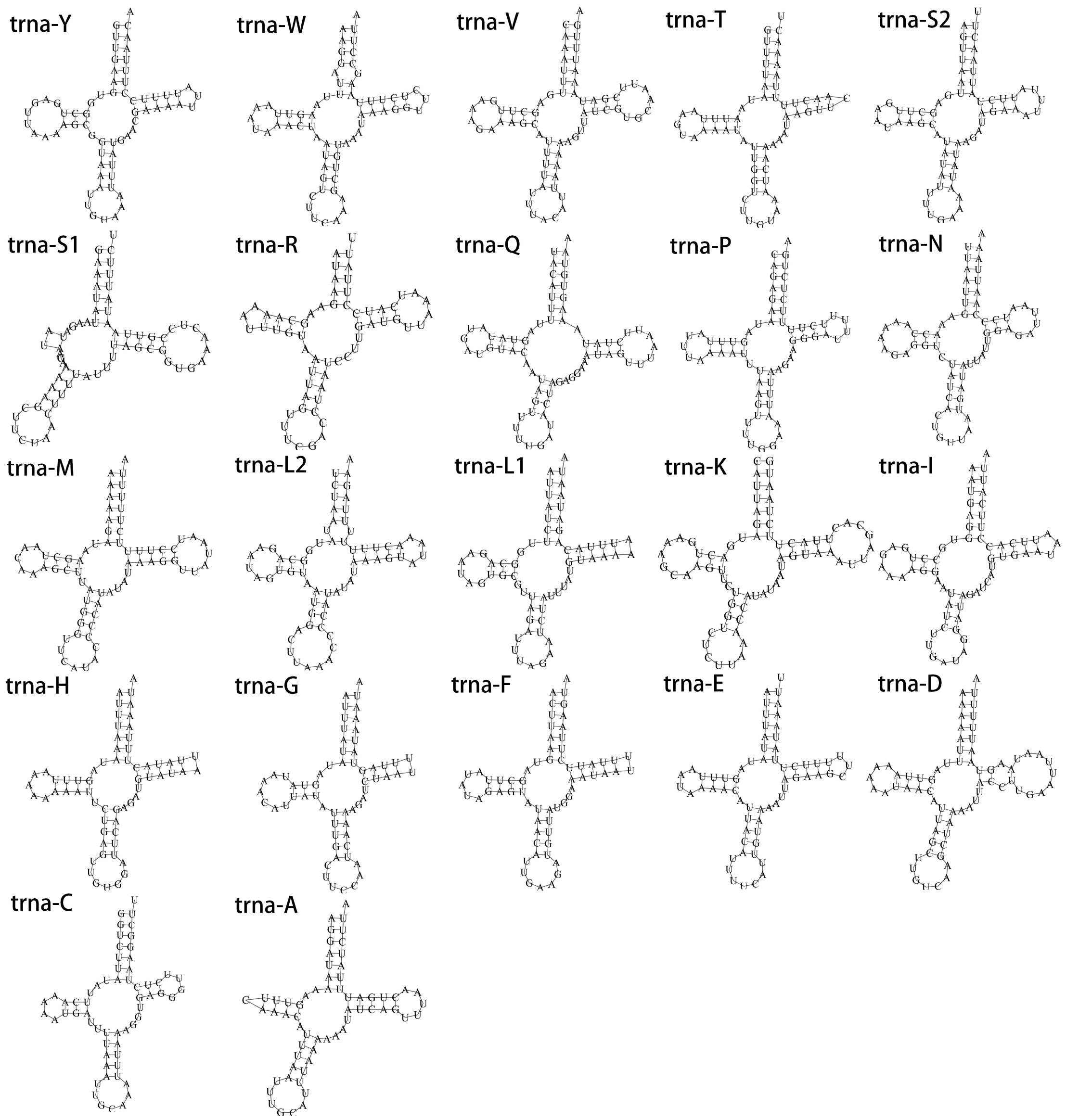

Figure S3. The secondary cloverleaf structure for the tRNAs of *Coomaniella dentata.*

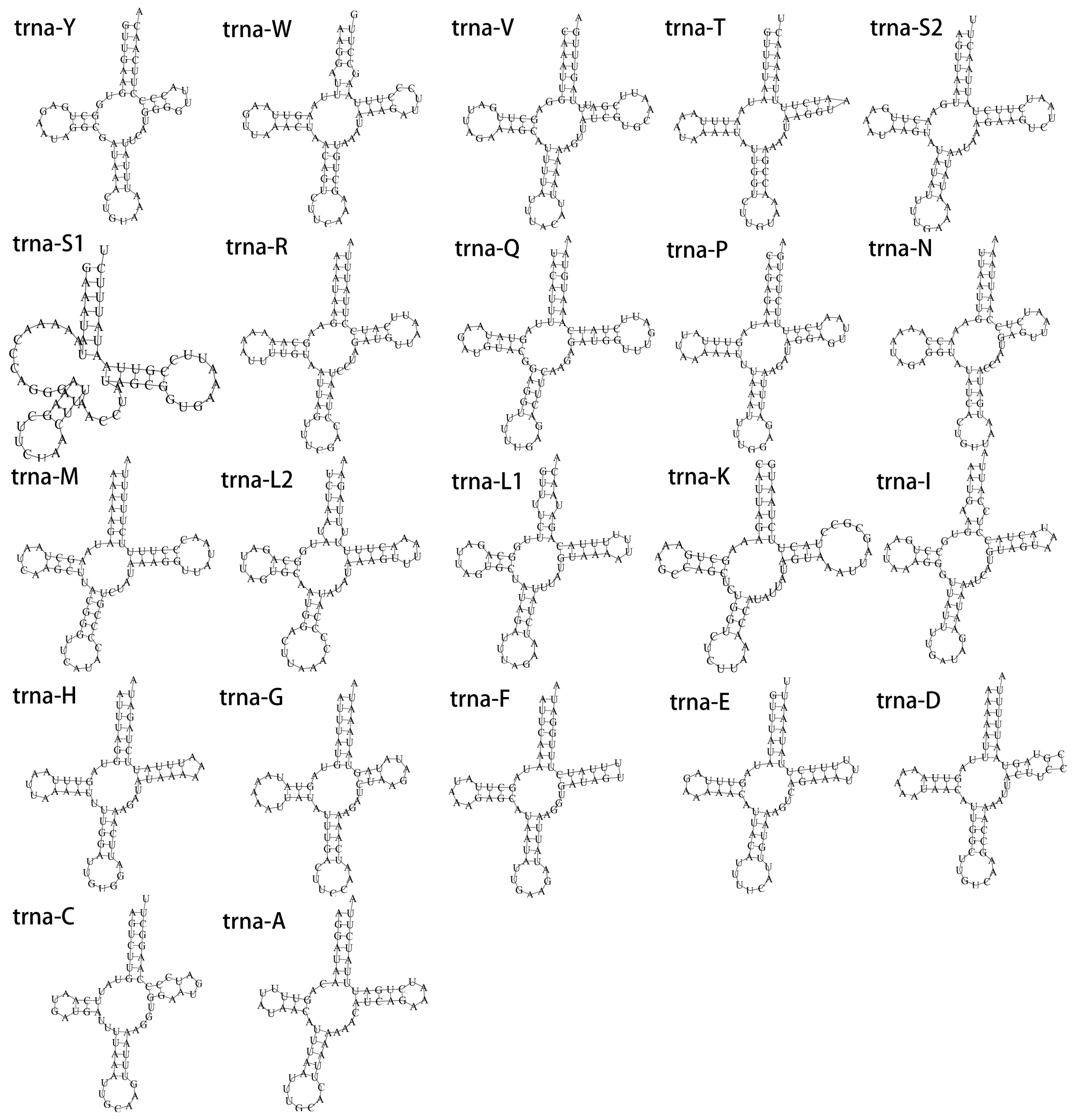

Figure S4. The secondary cloverleaf structure for the tRNAs of *Dicerca corrugata.*

Table S1. Codon usage of the protein-coding genes in *Coomaniella copipes*.

Codon	Count	RSCU	Codon	Count	RSCU	Codon	Count	RSCU	Codon	Count	RSCU
UUU(F)	291	1.6	UCU(S)	124	2.66	UAU(Y)	132	1.67	UGU(C)	28	1.65
UUC(F)	73	0.4	UCC(S)	18	0.39	UAC(Y)	26	0.33	UGC(C)	6	0.35
UUA(L)	321	3.23	UCA(S)	97	2.08	UAA(*)	0	0	UGA(W)	85	1.77
UUG(L)	35	0.35	UCG(S)	4	0.09	UAG(*)	0	0	UGG(W)	11	0.23
CUU(L)	118	1.19	CCU(P)	77	2.35	CAU(H)	51	1.46	CGU(R)	24	1.75
CUC(L)	23	0.23	CCC(P)	20	0.61	CAC(H)	19	0.54	CGC(R)	0	0
CUA(L)	91	0.92	CCA(P)	32	0.98	CAA(Q)	67	1.89	CGA(R)	29	2.11
CUG(L)	8	0.08	CCG(P)	2	0.06	CAG(Q)	4	0.11	CGG(R)	2	0.15
AUU(I)	326	1.7	ACU(T)	75	1.8	AAU(N)	152	1.63	AGU(S)	25	0.54
AUC(I)	57	0.3	ACC(T)	17	0.41	AAC(N)	35	0.37	AGC(S)	4	0.09
AUA(M)	203	1.78	ACA(T)	72	1.72	AAA(K)	80	1.52	AGA(S)	97	2.08
AUG(M)	25	0.22	ACG(T)	3	0.07	AAG(K)	25	0.48	AGG(S)	4	0.09
GUU(V)	90	2.13	GCU(A)	83	2.05	GAU(D)	55	1.51	GGU(G)	48	0.92
GUC(V)	13	0.31	GCC(A)	27	0.67	GAC(D)	18	0.49	GGC(G)	9	0.17
GUA(V)	55	1.3	GCA(A)	50	1.23	GAA(E)	69	1.75	GGA(G)	130	2.49
GUG(V)	11	0.26	GCG(A)	2	0.05	GAG(E)	10	0.25	GGG(G)	22	0.42

Table S2. Codon usage of the protein-coding genes in *Coomaniella dentata.*

Codon	Count	RSCU	Codon	Count	RSCU	Codon	Count	RSCU	Codon	Count	RSCU
UUU(F)	319	1.76	UCU(S)	123	2.62	UAU(Y)	139	1.76	UGU(C)	33	1.89
UUC(F)	44	0.24	UCC(S)	18	0.38	UAC(Y)	19	0.24	UGC(C)	2	0.11
UUA(L)	391	3.88	UCA(S)	95	2.03	UAA(*)	0	0	UGA(W)	87	1.81
UUG(L)	22	0.22	UCG(S)	1	0.02	UAG(*)	0	0	UGG(W)	9	0.19
CUU(L)	125	1.24	CCU(P)	73	2.23	CAU(H)	53	1.54	CGU(R)	21	1.56
CUC(L)	13	0.13	CCC(P)	12	0.37	CAC(H)	16	0.46	CGC(R)	2	0.15
CUA(L)	48	0.48	CCA(P)	46	1.4	CAA(Q)	69	1.89	CGA(R)	29	2.15
CUG(L)	5	0.05	CCG(P)	0	0	CAG(Q)	4	0.11	CGG(R)	2	0.15
AUU(I)	348	1.81	ACU(T)	90	2.07	AAU(N)	162	1.71	AGU(S)	28	0.6
AUC(I)	36	0.19	ACC(T)	16	0.37	AAC(N)	28	0.29	AGC(S)	3	0.06
AUA(M)	213	1.86	ACA(T)	67	1.54	AAA(K)	88	1.73	AGA(S)	100	2.13
AUG(M)	16	0.14	ACG(T)	1	0.02	AAG(K)	14	0.27	AGG(S)	7	0.15
GUU(V)	99	2.4	GCU(A)	81	2.1	GAU(D)	65	1.86	GGU(G)	44	0.87
GUC(V)	2	0.05	GCC(A)	17	0.44	GAC(D)	5	0.14	GGC(G)	4	0.08
GUA(V)	57	1.38	GCA(A)	54	1.4	GAA(E)	77	1.9	GGA(G)	137	2.7
GUG(V)	7	0.17	GCG(A)	2	0.05	GAG(E)	4	0.1	GGG(G)	18	0.35

Table S3. Codon usage of the protein-coding genes in *Dicerca corrugata.*

Codon	Count	RSCU	Codon	Count	RSCU	Codon	Count	RSCU	Codon	Count	RSCU
UUU(F)	238	1.41	UCU(S)	77	1.86	UAU(Y)	118	1.41	UGU(C)	30	1.62
UUC(F)	100	0.59	UCC(S)	31	0.75	UAC(Y)	49	0.59	UGC(C)	7	0.38
UUA(L)	268	2.72	UCA(S)	99	2.39	UAA(*)	0	0	UGA(W)	78	1.63
UUG(L)	81	0.82	UCG(S)	8	0.19	UAG(*)	0	0	UGG(W)	18	0.38
CUU(L)	114	1.16	CCU(P)	69	2.08	CAU(H)	46	1.31	CGU(R)	21	1.47
CUC(L)	19	0.19	CCC(P)	19	0.57	CAC(H)	24	0.69	CGC(R)	1	0.07
CUA(L)	90	0.91	CCA(P)	40	1.2	CAA(Q)	69	1.86	CGA(R)	25	1.75
CUG(L)	20	0.2	CCG(P)	5	0.15	CAG(Q)	5	0.14	CGG(R)	10	0.7
AUU(I)	289	1.57	ACU(T)	73	1.5	AAU(N)	140	1.51	AGU(S)	25	0.6
AUC(I)	80	0.43	ACC(T)	42	0.86	AAC(N)	45	0.49	AGC(S)	4	0.1
AUA(M)	174	1.63	ACA(T)	73	1.5	AAA(K)	69	1.38	AGA(S)	78	1.89
AUG(M)	40	0.37	ACG(T)	7	0.14	AAG(K)	31	0.62	AGG(S)	9	0.22
GUU(V)	96	1.89	GCU(A)	84	1.83	GAU(D)	55	1.47	GGU(G)	58	1.09
GUC(V)	20	0.39	GCC(A)	31	0.67	GAC(D)	20	0.53	GGC(G)	6	0.11
GUA(V)	74	1.46	GCA(A)	58	1.26	GAA(E)	58	1.55	GGA(G)	116	2.19
GUG(V)	13	0.26	GCG(A)	11	0.24	GAG(E)	17	0.45	GGG(G)	32	0.6

Table S4. Summarized A+T contents of 13 PCGs for the three buprestid species investigated in this study.

genes	A+T (%)		
	C. copipes	*C. dentata*	*D. corrugata.*
nad2	74.49	76.74	70.48
cox1	66.23	68.71	63.36
cox2	69.20	72.73	66.72
atp8	79.49	81.41	77.56
atp6	71.26	75.26	71.26
cox3	68.61	71.53	65.44
nad3	74.01	77.12	67.51
nad5	76.98	79.07	72.85
nad4	76.35	78.67	73.43
nad4l	77.89	77.19	74.23
nad6	79.80	80.78	75.25
cytb	69.82	71.83	66.75
nad1	74.42	75.97	71.40
